# Comparison between Preoperative Rectal Diclofenac Plus Paracetamol and Diclofenac Alone for PostoperativePain of Hysterectomy

**Published:** 2014-09

**Authors:** Saghar Samimi Sede, Fateme Davari Tanha, Mehrnaz Valadan, Zeinab Modaressi

**Affiliations:** 1 Department of anesthesiology, women Hospital, Tehran University of medical sciences, Tehran, Iran; 2 Department of obstetrics & gynecology, women Hospital, Tehran University of medical sciences, Tehran, Iran

**Keywords:** Postoperative Pain, Rectal Diclofenac, Paracetamol

## Abstract

**Objective:** To detect whether the preoperative combined administration of rectal diclofenac and paracetamol is superior to placebo or rectal diclofenac alone for pain after abdominal hysterectomy.

**Materials and methods:** Ninety female patients (American Society of Anesthesiologists (ASA) physical status I-II), scheduled for abdominal hysterectomy were recruited to this double blind trial and were randomized to receive one of three modalities before surgery: rectal combination of diclofenac and paracetamol, rectal diclofenac alone or rectal placebo alone which were given as a suppository one hour prior to surgery. The primary outcomes were visual analogue pain scores measured at 0, 0.5, 2, 4, 8, 16 and 24 hours after surgery and the time of first administration and also total amount of morphine used in the first 24 hour after surgery. A 10 cm visual analog scale (VAS ) was used to assess pain intensity at rest.

**Results:** In patients receiving the combination of diclofenac and paracetamol total dose of morphine used in the first 24 hour after surgery was significantly lower (13.9 ± 2.7 mg) compared to diclofenac group (16.8± 2.8 mg) and placebo group (20.1 ± 3.6 mg) (p<0.05). VAS pain score was significantly lower in combination group compared to other groups all time during first 24 hours (p<0.05). There had been a significant difference between combination group and the two other groups in terms of the first request of morphine (p<0.05).

**Conclusion:** According to our study Patients who receive the rectal diclofenac-paracetamol combination experience significantly a lower pain scale in the first 24 hour after surgery compared with patients receiving diclofenac or placebo alone. Their need to supplementary analgesic is significantly later and lower compared to placebo and diclofenac alone.

## Introduction

Postoperative pain is a major concern because it affects multiple systems and induces physiological, immunological, and psychologicalchages (1,2). Despite many advances in the provision o pain services, acute pain after surgery remains a serious causes of sever suffering that is often undermanaged despite our best efforts (3). Acute pain can be persisted, the tissue damage of surgery setting up pathophysiological processes in the peripheral and central nervous system that may produce chronicity (4). The association between surgery , acute pain and ongoing severe chronic pain is well defined (5,6). There is therefore a pressing need of advances in the agents and techniques we can use to improve analgesia efficacy, and perhaps reduce the incidence of chronic suffering after surgery. Pain management in the perioperative period has been traditionally based on opiates. Concidering their side effects (respiratory depression, nausea, vomitin and histamine release), new drugs, opiate-sparing drugs, and novel techniques were introduced for treatment. A moltimodal approach has been suggested to improve postoperative analgesia and to reduce the opioid- related side-effects (7). Non-opioids play an ever increasing role in the treatment of postoperative pain.Different classes of analgesics exert their effects through different mechanisms. A combination of analgesics from different classes may provide additive analgesic effects with fewer side effects than when a single therapeutic drug is used (8) .Paracetamol is frequently used as non opioid analgesic in postoperative pain and has a morphine-sparing effect .Its mechanism of action is not fully understood ,but it is generally accepted that paracetamol is a centrally acting drug (8). Diclofenac is a commenly used analgesic, that has analgesic , anti inflammatory properties and exerts its action via inhibition of prostaglandin synthesis by inhibiting cyclooxygenase-1 and cyclooxygenase-2 with relative equipotency (9). In clinical practice, NSAIDs and paracetamol are widely used together, but whether such a combination actually offers a significant improvement in analgesia is contraversial (10, 11). Both rectal diclofenac and paracetamol are commonly used to traet acute postoperative pain but combining them to improve the quality of analgesia is contraversial. This study aimed to detect whether the pre-operative combined administration of rectal diclofenac and paracetamol is superior to rectal diclofenac alone.

## Materials and methods

The protocol was approved by ethics and clinical studies committee of Tehran University of Medical Sciences and informed consent was obtained from all patients who were in study. This study is a prospective double blind, randomized, placebo controlled study, involving 90 women of ASA I, II, aged 40-65 BMI<40, undergoing elective abdominal hysterectomy in Women’s hospital (TUMS). Exclusion criteria were patient refusing, asthma, known allergy or contraindication to the study drugs, kidney or hepatic disfunction, haemorrhagic diathesis, and duration of surgery more than 2 hours. All patients were given a verbal explanation about the study including the use of visual analogue scale for pain assessment (0 as "no pain to 10 as "worst imaginable pain"). all the patients were pre-medicated with oral diazepam 0.2mg/kg given the night before the surgery. The patients were randomly assigned to 1 of the 3 treatment groups using a computer-generated table. All suppositories were identical-looking but with different gradients. A nurse who did not participate in the study was asked to administered 50 mg diclofenac suppository to group A, suppositories contained of 50 mg diclofenac plus 325 mg paracetamol to group B and placebo suppositories to group C (control group) 30 min prior to surgery, none of the patients was informed of type of drug prescribed. On arrival in the operating room, a crystalloid IV infusion was started, and baseline mean arterial blood pressure, heart rate, and peripheral oxygen saturation obtained using standard monitors. Anesthesia was induced with midazolam 0.05mg/kg, fentanyl 3µg/kg, propofol 2mg/kg and atracurium 0.5mg/kg. The operation was performed by the same surgeon. After intubation, ventilation was controlled to maintain normocapnia with isoflurane in %35 oxygen with nitrous oxide. During the maintenance, IV fentanyl was given intermittently as an analgesic when required, no more than 1µg/kg. The amount of the induction agent and volatile agent and opioid were titrated by the attending anesthetist, who was blind to patient’s group, and recorded. At the end of surgery residual neuromuscular blockade was antagonized with neostigmine 0.05mg/kg and atropine 0.02mg/kg. After extubation, patients were transferred to the post-anesthetic care unit, and their pain scores and opioid usage were measured and recorded on arrival and every 30 min until discharge from the PACU and at 4-,6-,8-,12-,18-,24 hours after end of surgery, by a research assistant blinded to group allocation. The patients were discharged back to the ward after 2 hours. When patients requested analgesia, or had VAS≥4 we provided using 2mg IV bolus injections of morphine. The incremental bolus dose of morphine was increased to 3 mg if analgesia was inadequate (VRS pain score ≥5) after the first hour of the last injection. Ondansetron 4 mg i.v was given as a rescue antiemetic if needed. The primary outcome measure of study was intensity of pain during 24 hours follow up period. Secondary outcome measure was total amount of morphine consumption.

***Statistical analysis***

Sample size estimation showed that 30 per treatment group would be adequate to detect a clinically relevant reduction of the level of pain by %25 with a power of 0.80 and a level of significance of %5.Statistic tests were performed using SPSS 13.0 for windows (SPSS 16, Chicago, IL, USA). Continuous variables were analyzed using student’s T-test. Nominal parametric data were analyzed using the fisher exact test or Mann-Whitney test. Results are reported as absolute value, means or numbers. A p value of <0.05 was considered significant.

## Results

All 90 patients completed the study. There were no significant differences between three groups with regard to patient’s age, sex, weight and duration of surgery (Table 1).

VAS shows that during the first 24 hours the patients in combination group had the lowest pain compared to the other two groups and also diclofenac group had less pain than placebo group with statistically significant difference between diclofenac-paracetamol group versus both other groups (p<0.05) and also diclofenac group versus placebo group (p<0.05) (Table 2).

Total dose of morphine was lowest in diclofenac-paracetamol group (13.9 ± 2.7 mg) which is then followed by diclofenac group (16.8 ± 2.8 mg) and placebo group (20.1 ± 3.6 mg) and there was a statistically significant difference between combination group versus two other groups (p<0.05) (Table 3). There had been a significant difference between combination group and the two other groups in terms of the first request of morphine (p<0.05).

## Discussion

The results demonstrate that pre-operative administration of rectal diclofenac and paracetamol reduced the need for postoperative morphine during the first 24 hours. Patients who received both drugs recorded a lower pain rating scale.

**Table 1:** Patient’s demographic data and time of operation

	**Diclofenac-paracetamol** **n=30**	**Diclofenac** **n=30**	**Placebo** **n=30**	**p value**
Weight (kg) mean (SD)	69 ± 10	67.2 ± 8	68 ± 8	0.9
Age (yr) mean (SD)	53.4 ± 1.5	55.9 ± 1.11	54 ± 1.4	0.8
Surgery duration (min) mean (SD)	106 ± 2.67	109 ± 1.54	106±1.12	0.3
Height mean (SD)	160.2 ±1.1	162.5 ±2.1	159.4 ±	0.45

p value< 0.05 is significant.

**Table 2:** Incidence of VAS≥ 4 after anesthesia

**Postoperative time** **(hour)**	**Diclofenac-paracetamol** **n (%)**	**Diclofenac** **n (%)**	**Placebo** **n (%)**
0	0	0	0
0.5	0*	1 (3%)* ®	7 (23%) ®
2	0* Ɨ	6 (20%)*	10 (32%)
4	5 (16%)* ®	9 (30%)*	13 (43%)
8	8 (22%)* Ɨ	11 (36%)*	16 (53%)
16	11 (36%)* Ɨ	16 (53%)*	20 (66%)
24	12 (40%)* Ɨ	16 (53%)*	21 (67%)

* p<0.05 versus placebo;

Ɨ p<0.05 against diclofenac;

® Beginning of morphine administration

**Table 3:** Total morphine requirement

	**Diclofenac-paracetamol** **mean± SD**	**Diclofenac** **mean± SD**	**Placebo** **mean± SD**
Total morphine consumption (mg)	13.9 ±2.7* Ɨ	16.8 ±2.8*	20.1±3.6

* p<0.05 against placebo;

Ɨ p<0.05 against diclofenac

Many studies have been done on this issue, with different results. In Romsing et al (12) met analyzed 6 other studies that compared analgesic effects of NSAID + paracetamol with NSAID alone, only two studies showed that pain scores were significantly reduced after administration of the combination of the NSAID and paracetamol compared with the NSAID alone (13, 14) also only in 3 studies this combination was began prior to surgery (15, 16, 17), Montgomery and colleagues (17) reported that the use of this combination has been shown to reduce the amount of morphine required for adequate analgesia postoperatively by about one-third compared with paracetamol alone in women undergoing elective abdominal gynecological procedures. The time to first administration of analgesic was recorded in only two of these studies (14, 16). Only one of these studies showed that combination therapy has increased time to the first administration of analgesic (14). In our study combination therapy resulted in lengthening of time to the first administration of analgesic and reduction in pain scores. In Munishankar and colleague’s study, the studied drugs were administrated after surgery and required dosage of morphine was not significantly different between two groups of patients (18). In our study the drugs were given 1h prior to surgery and needed amount of morphine was significantly reduced in combination therapy group. In our study the most significant pain reduction occurred at early hours after surgery, when the pain is at its peak. This can be attributed to peak plasma concentration of administered drugs (diclofenac and paracetamol), that after rectal administration (prior to surgery), reach their peak plasma concentration after 2-3h, therefore explaining their peak clinical effect in 4 hours after administration. In Moussa and Riad study the effect of diclofenac alone or diclofenac- paracetamol was compared (19). The study was undergone in children and the drugs were administrated before operation, and it showed effective reduction of pain with combination therapy, which was similar to our study, but our patients were adult women.

## Conclusion

The present findings indicate that administration of the combination of rectal paracetamol and diclofenac reduces pain intensity and total amount of morphine usage and also results in lengthening of the first administration of analgesic compared to rectal diclofenac alone or placebo. According to our study, patients receiving the rectal diclofenac- paracetamol combination experienced significantly a lower pain scale in first 24-hour after surgery as compared with patients receiving diclofenac or placebo alone. Their need to supplementary analgesic was significantly later and lower as compared to placebo and diclofenac alone.

## References

[1] Holte K, Kehlet H (2002). Effect of postoperative epidural analgesia on surgical outcome. Minerva Anestesiol.

[2] Kiecolt-Glaser JK, Page GG, Marucha PT, MacCallum RC, Glaser R (1998). Psychological influences on surgical recovery. Perspectives from psychoneuroimmunology. Am Psychol.

[3] Apfelbaum JL, Chen C, Mehta SS, Gan TJ (2003). Postopertative pain experience: result from a national survey suggest postoperative pain continues to be undermanaged. Anesth Analg.

[4] Cousins MJ, Power I, Smith G (2000). Pain_a persistent problem. Reg Analg Pain Med.

[5] Macrae WA (2001). Chronic pain after surgery. Br J Anesth.

[6] Perkins FM, Kehlet H (2000). Chronic pain as an outcome of surgery. A review of predictive factors. Anesthesiology.

[7] White PF (2005). The changing role of non-opioid analgesic techniques in the management of postoperative pain. Anesth Analg.

[8] Schug SA, Manopas A (2007). Update on the role of non-opioids for postoperative pain treatment. Best Pract Res Clin Anaesthesiol.

[9] Cobby TF, Crighton IM, Kyriakides K, Hobbs GJ (1999). Rectal paracetamol has a significant morphine-sparing effect after hysterectomy. Br J Anaesth..

[10] Gottschalk A, Ochroch EA (2003). Preemptive analgesia:What do we do now?. Anesthesiology.

[11] Merry A, Power I (1995). Perioperative NSAIDs: towards greater safety. Pain Rev.

[12] Romsing J, Moiniche S, Dahl JB (2002). Rectal and parenteral paracetamol, and paracetamol in combination with NSAIDs, for postoperative analgesia. Br J Anesth.

[13] Fletcher D, Nègre I, Barbin C, François A, Carreres C, Falgueirettes C (1997). Postoperative analgesia with i.v. propacetamol and ketoprofen combination after disc surgery. Can J Anaesth.

[14] Breivik EK, Barvoll P, Skovlund E (1999). Combination diclofenac with acetaminophen or acetaminophen-codeine after oral surgery: a randomized, double-blind single-dose study. Clin Pharmacol Ther.

[15] Morton NS, O’Brien K (1999). Analgesic efficacy of paracetamol and diclofenac in children recieving PCA morphine. Br J Anaesth.

[16] Van Lancker P, Vandekerckhove B, Cooman F (1999). The analgesic effect of preoperative administration of propacetamol, tenoxicam or a mixture of both in arthroscopic, outpatient knee surgery. Acta Anaesthesiol Belg.

[17] Montgomery JE, Sutherland CJ, Kestin IG, Sneyd JR (1996). Morphine consumption inpatients receiving rectal paracetamol and diclofenac alone and in combination. Br J Anaesth.

[18] Munishankar B, Fettes P, Moore C, McLeod GA (2008). A double-blind randomised controlled trial of paracetamol, diclofenac or the combination for pain relief after caesarean section. Int J Obstet Anesth.

[19] Riad W, Moussa A (2007). Pre-operative analgesia with rectal diclofenac and/or paracetamol in children undergoing inguinal hernia repair. Anaesthesia.

